# Genetic and Genomic Approaches to the Study of Drug‐Induced Liver Injury

**DOI:** 10.1111/liv.16191

**Published:** 2024-12-20

**Authors:** Ann K. Daly

**Affiliations:** ^1^ Faculty of Medical Sciences, Translational & Clinical Research Institute Newcastle University Newcastle upon Tyne UK

**Keywords:** amoxicillin‐clavulanate, flucloxacillin, genetics, genomics, human leucocyte antigen, idiosyncratic drug‐induced liver injury, isoniazid

## Abstract

Idiosyncratic hepatotoxicity induced by prescribed drugs has been known since the early 20th century. Identifying risk factors, including genetic factors, that trigger this drug‐induced liver injury (DILI) has been an important priority for many years, both to prevent drugs that cause liver injury being licensed and as a potential means of preventing at‐risk patients being prescribed causative drugs. Improved methods for genomic analysis, particularly the development of genome‐wide association studies, have facilitated the identification of genomic risk factors for DILI, but, to date, there are only two main examples, liver injury caused by amoxicillin‐clavulanate (AC) and by flucloxacillin, where genetic risk factors causing the injury have been identified and replicated with understanding of the underlying mechanism. There has also been progress on identifying genetic risk factors for liver injury caused by other anti‐infective agents, herbal remedies and nonsteroidal anti‐inflammatory drugs. The majority of genetic risk factors identified to date are specific human leucocyte antigen (HLA) alleles and evidence that these alleles preferentially present self‐peptides inappropriately to T cells in the liver has been obtained. Non‐HLA genes also contribute to genetic susceptibility, both as co‐factors in T‐cell responses and, in the case of isoniazid‐only, drug metabolism. Polygenic risk scores to predict DILI have been developed, both a simple score that predicts AC injury and complex scores that may be applied to DILI more generally and provide evidence that additional risk factors other than HLA genes exist.

**Summary**

- Idiosyncratic drug‐induced liver injury (DILI) can be triggered by a range of compounds, including anti‐infectives, nonsteroidal anti‐inflammatory drugs, herbal medicines, monoclonal antibodies and mRNA vaccines.
- Specific human leucocyte antigen alleles represent the most common genetic risk factors for the development of DILI, but other immune response genes and drug metabolism genes may also contribute.
- Amoxicillin‐clavulanate and flucloxacillin are both common inducers of liver injury with well‐replicated genetic risk factors now identified. Low positive predictive values have prevented routine adoption in the clinic.
- Additional genetic risk factors for liver injury due to other drugs need further investigation.

**Abbreviations**
AC: amoxicillin‐clavulanate
DILI: drug‐induced liver injury
GWAS: genome‐wide association study
HILI: herbal medicine‐induced liver injury
HLA: human leucocyte antigen
MHC: major histocompatibility complex
NAT2: N‐acetyltransferase 2
NSAID: nonsteroidal anti‐inflammatory drug
PRS: polygenic risk score
SNPs: single‐nucleotide polymorphisms
TB: tuberculosis

## 1 Introduction

Drug‐induced liver injury (DILI) remains an important concern for both hepatologists and drug developers. Although many drugs show predictable concentration‐dependent hepatotoxicity that is relatively easy to detect in non‐clinical studies, other licensed drugs can cause idiosyncratic DILI which is more difficult to detect and, because of its relative rarity, may not emerge until after the drug has been licensed. This has led to a number of high‐profile drug withdrawals post‐registration but, for other drugs, the rarity of idiosyncratic DILI and the benefits associated with the individual drug mean it continues to be licensed. Idiosyncratic DILI is not simply associated with small molecule therapeutics; the possibility of DILI reactions to herbal medicines has also been appreciated for many years. Recently, cases of DILI due to more novel medicines have emerged, with biologic agents now an important contributor as well as some evidence that mRNA vaccines can show rare hepatotoxicity [1, 2]. It is considered that the effects seen with biologics and related drugs may be more appropriately classed as indirect hepatotoxicity not idiosyncratic, as discussed in more detail in Section 3.7 [1].

Despite the advances made in genomics generally in the past 25 years and the considerable ongoing efforts to apply these to DILI, susceptibility to DILI remains difficult to predict. As summarised in Table 1 and discussed in more detail below, there are two main drugs causing DILI where there has been a clear demonstration of significant genetic risk using a genome‐wide association study (GWAS), followed by independent replication and mechanistic studies. Genetic susceptibility clearly extends beyond these two specific examples, but replication of other findings is more limited either due to the lack of replication cohorts or because the replication study has not confirmed the original finding. At present, there are no regulatory recommendations for using genetic tests to predict DILI susceptibility prior to drug prescription, despite a number of advances in understanding the contribution of certain genes, especially human leucocyte antigen (HLA) genes, to risk. In this article, our current knowledge of genetic risk factors for DILI and their interaction with non‐genetic risk factors is reviewed in detail together with prospects for further advances.

**TABLE 1:** Genetic risk factors for DILI that have been replicated with some mechanistic data also available.

Drug	Gene	Allele	Allele frequency in European DILI cases	Allele frequency in European controls	Odds ratio (allelic) and *p* value (from GWAS)	Comments	References
Amoxicillin‐clavulanate	*HLA‐DRB1*	DRB1*15:01	0.30	0.12	3.14 (3.66–2.69); 1.4 × 10^−47^	Reported originally in two candidate gene studies; replicated in 133 case‐cohort post‐GWAS; individual CD4‐positive T‐cell clones reactive to amoxicillin and clavulanate isolated from one AC DILI patient positive for this variant	[3, 4]
	*HLA‐A*	A*02:01	0.44	0.28	2.11 (2.42–1.84); *p* = 4.5 × 10^−26^	Replicated in 133 case‐cohort post‐GWAS; CD4‐positive T‐cell clones reactive to amoxicillin and clavulanate isolated from 2 AC DILI patients positive for this variant	[3, 4]
	*HLA‐B*	B*15:18	0.01	0.004	3.1 (6.08–1.58); *p* = 0.001	Replicated in 133 case‐cohort post‐GWAS	[4]
	*PTPN22*	rs2476601	0.13	0.08	1.62 (1.32–1.98); 4.0 × 10^−6^	Variant is a risk factors for the development of several autoimmune diseases; replicated in 133 case‐cohort post‐GWAS	[4, 5]
	*ERAP2*	rs1363907	0.67	0.58	1.68 (1.23–1.66); 1.7 × 10^−7^	Replicated in 133 case‐cohort post‐GWAS	[4]
Flucloxacillin	*HLA‐B*	B*57:01	0.42	0.04	36.6 (26.1–51.3); *p* = 2.6 × 10^−97^	Original report involving 51 cases replicated in a second cohort of 23 cases; CD8‐positive T‐cell clones isolated from four flucloxacillin DILI cases positive for B*57:01	[6, 7, 8]

*Note:* Data reported here are entirely for White Europeans due to negligible data available to date for other ethnicities on genetic risk factors for DILI due to these two drugs.

Abbreviations: GWAS, genome‐wide association study; HLA, human leucocyte antigen.

## 2 Approaches to Studying Dili and Genetic Risk Factors

As reviewed by Zimmerman [9], who pioneered DILI research, idiosyncratic hepatotoxicity due to prescribed drugs has been an issue since the early 20th century. Early reports include accounts of liver toxicity due to cinchophen, a nonsteroidal anti‐inflammatory (NSAID) drug together with sulfonamides and antithyroid agents [9]. An increase in the range of drugs available for prescription from the 1950s onwards also coincided with an increased number of reports of DILI [9]. It was well recognised then that these reactions were relatively rare and idiosyncratic, but data on precise frequency and whether the observed idiosyncrasy related to patient genetics were not available. The issue of low frequency remains, but the development of national and international reporting systems for adverse reactions has assisted greatly in our current understanding of the frequency of some of these reactions.

The traditional approach to understanding genetic variation generally has been to perform twin and family studies [10], but applying these approaches to DILI or other rare adverse drug reactions is challenging, especially as family members are generally not exposed to the same causative drugs. A few family studies on DILI have been reported with the earliest of these concerned with phenytoin and involving studies in vitro [11]. Lymphocytes from patients with phenytoin DILI showed dose‐dependent toxicity when exposed to phenytoin metabolites as did cells from some parents and siblings which led to the conclusion that toxicity was inherited. A further study followed up a family where three siblings had suffered hepatitis following phenytoin prescription [12]. Again, lymphocytes from some but not all siblings naive to phenytoin showed similar toxicity to the drug‐exposed individuals. A broadly similar approach was taken independently in studies on amineptine, a tricyclic antidepressant known to cause DILI [13]. These early studies all suggested that DILI might be predictable based on patient genetics but provided limited insights into which genes should be studied.

Though such twin/family studies represent the traditional approach for studying monogenic diseases that arise due to a single gene defect, progress in recent years in understanding polygenic diseases, where a number of different genetic risk factors contribute, has been mainly as a result of case–control studies where frequencies of selected genetic polymorphisms are compared between confirmed cases of the disease and unaffected controls using unrelated individuals. Originally, such case–control studies involved studying selected polymorphisms in candidate genes likely to be relevant to the disease. It was considered that DILI might not be a monogenic disease and, in parallel with the family studies mentioned above, studies using a candidate gene case–control approach were initiated with the earliest of these examples isoniazid‐related DILI. Isoniazid is known to cause DILI in 1%–5% of patients undergoing treatment [14]. Isoniazid metabolism has been well studied in part because a polymorphism in N‐acetyltransferase 2 (*NAT2* polymorphism) was detected in the 1950s when isoniazid was first used to treat tuberculosis (TB), with acetylation shown to be a key step in its metabolism [15]. Approximately 50% of Europeans are fast acetylators and metabolise isoniazid more rapidly than the remaining approx. 50% who are termed slow acetylators. In the 1970s, DILI due to isoniazid began to attract more concern and the possibility that acetylator status might be a predictor of this toxicity was investigated in detail for the first time, though the early studies did not give clear findings [16, 17]. Another early pharmacogenetic study relevant to DILI concerned the debrisoquine polymorphism (now termed the *CYP2D6* polymorphism) where patients who had suffered hepatotoxicity due to perhexiline appeared more likely to be debrisoquine‐poor metabolisers [18].

In parallel with these candidate gene pharmacogenetic studies, HLA serotype was also investigated as a determinant of DILI susceptibility. Other liver diseases such as autoimmune hepatitis had been shown to have HLA associations, and many DILI cases showed hypersensitivity features, which made it likely that HLA serotype might be relevant to risk. A few reports suggested an association between the HLA serotype DR2 and halothane‐related DILI [19, 20]. A study of DILI cases associated with several drugs including nitrofurantoin found an apparent increased frequency of the HLA class II serotypes HLA‐DR2 and HLA‐DR6 [21]. A larger study involving several causative drugs found a trend towards significance for the class I serotype HLA‐A11 in DILI associated with tricyclic antidepressants and diclofenac and for the class II serotype HLA‐DR6 with DILI due to chlorpromazine [22]. These early reports have limitations due to HLA serotyping capturing overall HLA diversity less well than genotyping. Two further candidate gene studies on HLA associations with DILI used genotyping rather than serotype determination and were concerned with amoxicillin‐clavulanate (AC)‐related DILI. Both independent studies reported an identical association with the *HLA*‐DRB1*15:01 allele, which corresponds to the DR2 serotype mentioned above [23, 24].

These initial HLA genotyping studies at the start of the 21st century were followed by further genotyping studies involving non‐HLA genes as DILI candidate risk factors. The focus was mainly on pharmacogenetic risk factors relating to drug disposition but also on oxidative stress genes. Although a number of positive associations have emerged from a relatively large number of studies using this candidate gene approach (reviewed by Daly [25]), many represent single reports on small numbers of cases with no independent replication of the individual findings so need to be treated with caution. Once genotyping techniques that allowed genotype for many different polymorphisms throughout the genome to be determined became available, these GWAS studying large numbers of both cases and controls (> 1000 of each), led to the detection of genetic risk factors for polygenic diseases including type II diabetes mellitus, cancer and various metabolic diseases. As discussed below, progress has also been made in identifying risk factors for DILI using GWAS and this has facilitated the development of polygenic risk scores (PRS).

Interest in genetic risk factors for DILI has continued and has been facilitated by increased understanding of the human genome, the development of improved approaches such as use of GWAS and collaborative studies involving collection of larger numbers of DILI cases. GWAS approaches have now generally replaced candidate gene case–control studies as a means of identifying genetic risk factors for DILI.

## 3 Dili Genetic Risk Factors for Individual Drugs or Drug Classes

There is considerable phenotypic heterogeneity within idiosyncratic DILI. Although the term DILI encompasses a range of different phenotypes including hepatitis and cholestasis but also drug‐induced steatosis, fibrosis and veno‐occlusive disease (reviewed by Kaplowitz [26]), this section will consider only the three most common idiosyncratic DILI phenotypes on which most genetic studies have focussed namely hepatocellular injury, cholestatic injury and mixed hepatocellular/cholestatic injury. Individual drugs predominantly cause DILI with one of these phenotypes, though there are some exceptions to this. In this section, genetic associations with particular drug causes of DILI are considered in detail. Sections 3.1, 3.4 deal specifically with anti‐infective agents mainly because of their importance as causes of DILI and reflect the good overall progress to date in understanding genetic risk factors for DILI due to these agents, particularly AC and flucloxacillin. Progress in replicating genomic data and understanding underlying mechanisms for DILI due to AC and flucloxacillin is more advanced than for other drug causes (Table 1). NSAIDs (Section 3.5) represent another drug class important for idiosyncratic DILI, though progress on understanding genetic risk factors for NSAID DILI has been more limited. DILI due to herbal medicines (Section 3.6) is of increasing importance and a particular common genetic risk factor has now been identified. Though not idiosyncratic DILI as such [1], genetic data on certain forms of indirect hepatotoxicity are now available and will be considered in Section 3.7. Few ‘general’ risk factors for DILI have emerged to date, but a few recent examples of more general, less drug‐specific, risk factors for idiosyncratic DILI will be considered separately in Section 3.8. A variety of other drugs, including statins, antivirals, anticonvulsants and antidepressants, also cause DILI but, due to very limited progress on genetic risk factors for these types of DILI, will not be considered further.

### 3.1 Amoxicillin‐Clavulanate DILI

As the most common cause of DILI in a number of population surveys in both Europe and North America [1, 27, 28, 29, 30], AC seems likely to be the most common cause of DILI worldwide. AC DILI typically presents as cholestatic or mixed DILI 1–4 weeks after the drug has been discontinued. It was thought originally that clavulanic acid was the main hepatotoxic component, partly because amoxicillin alone can also cause DILI, but at a considerably lower frequency than seen with AC. However, in vitro data on T‐cell responses (Section 4) indicate that both drugs contribute to the toxicity [3]. The high frequency of AC DILI among DILI cases generally has facilitated detailed studies on genetic susceptibility to this form of DILI, including the early HLA genotyping studies on two separate case sets described in Section 2 which showed a role for DRB1*15:01 in susceptibility [23, 24]. The contribution by this HLA class II allele to risk was confirmed by a larger GWAS of 201 cases [31]. The larger sample size and use of GWAS enabled a second HLA class I allele A*02:01 to be demonstrated as another independent risk factor. Further enlargement of this European cohort to 444 cases reconfirmed the two HLA risk factors but also identified rs2476601 in the *PTPN22* gene on chromosome 1 as a risk factor for DILI, both AC‐induced and also induced by some other drugs (Table 1 and Section 3.4) [32]. The enlarged case‐cohort also provided evidence for the existence of additional risk factors, another HLA class I allele HLA‐B*15:18 and rs1363907 in the *ERAP2* gene, which contributes to HLA antigen processing [4]. A PRS involving the five independent genetic risk factors for AC‐DILI was developed. Using a validation cohort with 133 new cases, all five risk factors and value of the PRS in predicting AC DILI susceptibility were confirmed [4]. In particular, more than 40% of AC DILI cases had PRS of 2 or higher compared with 10% of population controls and, based on prescribing rates and incidence AC DILI, a risk of 1 in 400 for development of AC DILI in those with a score of 2 or higher. The PRS and the HLA associations are very specific for AC DILI and can be used as a means of confirming the causative drug [4]. Future use in clinical settings to confirm AC DILI may also be feasible.

Almost all genetic studies on AC DILI to date have focussed on White Europeans with limited coverage of African Americans and Latin Americans. In general, findings on White Europeans have been confirmed for these two other ethnic groups [4] but there is a need for further studies to include people from Asia and Africa. An interesting insight into ethnic variation is provided by a report of AC DILI in a patient of South Asian ethnicity positive for the HLA allele DRB1*15:02 [33]. This additional DRB1*15 variant is extremely rare in Europeans but relatively common in South Asians and also East Asians. The PRS for AC DILI described above may benefit from extension to involve a more global focus as further genetic data become available.

### 3.2 Flucloxacillin DILI

In contrast to the AC example (Section 3.1), flucloxacillin prescribing is less universal worldwide and currently occurs mainly in certain European countries (Sweden, Ireland, UK, Portugal, Netherlands, Belgium, Luxembourg) [34] and Australasia. Presentation of flucloxacillin DILI is similar to that for AC DILI, with cases typically showing DILI with a cholestatic or mixed phenotype 1–4 weeks after the drug has been discontinued. Surveys in countries where this drug is prescribed suggest it is an important cause of DILI, with a Swedish study reporting it as the most common cause there between 1970 and 2004 [35]. Smaller regional studies in countries where AC is used more widely than in Sweden suggest levels of DILI caused by flucloxacillin are slightly higher than those for AC DILI [36, 37]. The most detailed pharmacoepidemiological studies on flucloxacillin DILI used data from the UK‐based Clinical Practice Research Datalink primary care database. The initial study reported an incidence of 8.5 per 100 000 for DILI within 45 days of prescription of flucloxacillin [38]. This incidence was confirmed in a further study which also reported that the risk for developing flucloxacillin DILI increased with age with those above 70 years showing an incidence of 45 per 100000 [39].

Studies on genetic susceptibility to flucloxacillin DILI were initiated only after 2001 approximately, enabling GWAS approaches to be implemented from the start. The earliest GWAS concerned 51 UK‐based European cases of flucloxacillin DILI. This study reported a highly significant association with the HLA class I allele B*57:01 with 84% of cases carrying the risk allele; this was replicated in a smaller group of additional cases [6]. A follow‐up study in a larger patient group (*n* = 197), which included cases from other countries where this drug is prescribed, found 83% of cases carried either HLA‐B*57:01 or the homologous but rare HLA‐B*57:03 allele [7]. Unlike the findings for AC DILI (Section 3.1 and Table 1), no non‐HLA alleles were identified as risk factors. As discussed in Section 4, the strong B*57:01 association is consistent with an inappropriate T‐cell response being triggered by drug‐modified peptides [8] and seems likely to involve a similar mechanism to that underlying AC DILI, despite the genetic risk alleles being different.

Though the B*57:01 association with flucloxacillin DILI is strong, approximately 15% of cases are not positive for any B*57 allele. Attempts to identify alternative genetic risk factors in these cases have been unsuccessful to date [7]. To date, studies on flucloxacillin DILI have been on White Europeans only so the absence of genetic studies on those of non‐European ethnicity is a limitation.

The possibility that B*57:01 genotyping could be implemented clinically either to prevent B*57:01 carriers being prescribed flucloxacillin or to diagnose flucloxacillin DILI when a patient presents with symptoms has been considered by several studies. Genotyping prior to prescription is not cost‐effective on the basis of several independent analyses showing a low positive predictive value [39, 40, 41] but using it as a diagnostic tool to help confirm a DILI diagnosis and the causative drug is more promising. The level of risk for B*57:01 carriers for developing flucloxacillin DILI is of a similar order of magnitude to those with an ‘at‐risk’ score for AC DILI via the PRS described in Section 3.1 for which diagnostic utility has already been demonstrated on a limited scale [4].

### 3.3 DILI Due to Anti‐Tuberculosis Drugs

Anti‐TB drugs are an important cause of DILI worldwide. The most important causative agent in the standard drug regimen of isoniazid, rifampicin, pyrazinamide and ethambutol is isoniazid, but there is also evidence that pyrazinamide alone can cause DILI [42]. In addition, several studies suggest that an isoniazid‐rifampicin combination is more hepatotoxic than isoniazid alone [42]. As described in Section 2, genetic studies on isoniazid DILI were the earliest such studies on DILI due to any causative drug and a large number of reports on various genetic associations for anti‐TB DILI, almost always involving candidate gene studies, continue to appear. The more recent of these generally concern DILI reactions from the standard dosing regimen of the four drugs described above, but there are also some studies on DILI due to isoniazid only or cases of DILI where the four‐drug regimen was used, but there is direct evidence that the causative drug is isoniazid.

The most widely investigated genetic risk factor for the development of isoniazid‐related liver injury has been the *NAT2* gene, which encodes NAT2, the major metabolising enzyme for isoniazid. Worldwide, between 10% and 60% of individuals lack this enzyme activity [43]. As reviewed by Richardson and colleagues [44], there is an extensive literature suggesting that NAT2 deficiency or slow acetylation is associated with an increased risk of developing isoniazid‐related DILI but not all reports agree on this with a few studies, especially on White Europeans, suggesting no effect. A recent meta‐analysis exploring 18 different studies on *NAT2* genotype as a predictor for anti‐TB DILI is also consistent with an increased risk in slow acetylators, especially for the subgroup of slow acetylator genotypes classed as ‘ultraslow acetylators’ [45]. The biological basis for the increased risk of DILI in slow or ultraslow acetylators is in line with the hypothesis that the toxic isoniazid metabolite hydrazine is more likely to accumulate in the absence of the NAT2 enzyme (Section 4) [46, 47]. Levels of the parent drug are also likely to be higher in these individuals which has recently been generally confirmed in studies based in South Africa and South America featuring both phenotyping and genotyping, though with the caveat that NAT2 allele patterns in these two populations are different to those in Europe and South Asia where most previous studies on isoniazid DILI have been based [48, 49]. Other candidate genes for anti‐TB DILI, including HLA genes and those relevant to drug metabolism, have also been investigated (reviewed by Bao et al. [50]), but most of these reports have not been well confirmed.

Despite the large number of candidate gene studies reported to date on anti‐TB DILI which suggests that large patient cohorts could be available for study by GWAS, there have been only 3 GWAS reported to date. These concerned an Ethiopian population [51], a Thai population [52] and combined White European and South Asian cases [53]. The Ethiopian study involved patients being treated with rifampicin only, making it different to other published studies, and also did not detect any genome‐wide significant signals so will not be discussed further here. The study in Thailand involved 79 cases of anti‐TB DILI in patients undergoing treatment with the standard four‐drug regimen and found a genome‐wide significant signal for the *NAT2* gene. Though most of the cases had relatively mild DILI which did not meet the recommended three‐ to fivefold above upper limit of normal of alanine aminotransferase for drug treatment interruption [14], the *NAT2* signal detected is consistent with what had been detected previously in the earlier candidate gene studies. In addition, there was no evidence of a signal in the HLA region. The most recent GWAS involved 70 White European and 55 Indian anti‐TB DILI cases [53]. These cases underwent expert adjudication by hepatologists in line with international recommendations [54]. All cases had suffered moderate to severe DILI with many treated with the standard four‐drug regimen, though some cases in the European cohort (61%) had developed DILI when treated with isoniazid only. A separate analysis of both cohorts resulted in a genome‐wide significant signal (rs117491755 in *ASTN2*) being detected in Europeans but not the Indian group [53]. Trans‐ethnic meta‐analysis of the GWAS did not result in any genome‐wide significance. HLA genotypes were imputed to investigate this gene region in detail, and the haplotype HLA‐B*52:01‐C*12:02 was found to show major histocompatibility complex (MHC)‐level significance in European cases. The Indian cohort alone did not show MHC‐level significance, but trans‐ethnic meta‐analysis showed significance for HLA‐B*52:01. The B*52:01 signal was for combined drug regimens and failure to see this with isoniazid‐only cases could indicate a role for HLA only in either pyrazinamide DILI or when rifampicin is also used. Though GWAS on either cohort separately or trans‐ethnic meta‐analysis failed to detect a genome‐wide significant *NAT2* signal similar to that seen in the Thai study [52], assignment of *NAT2* genotypes from the GWAS data found enrichment of the allele NAT2*6 among cases in the meta‐analysis and a significantly increased odds ratio for development of DILI in the European cohort in those individuals homozygous for NAT2*6 who can be referred to as ‘ultraslow acetylators’ [53]. This conclusion is consistent with that from the 18 study meta‐analysis discussed above [45].

In summary, current understanding of genetic risk factors for anti‐TB DILI is incomplete but a major role for HLA seems unlikely. The *NAT2* association has been confirmed using a number of different approaches, but further larger studies are still needed to identify additional genetic risk factors and also to confirm the precise *NAT2* genotypes at risk of DILI. Interethnic variation in patterns and frequencies of *NAT2* alleles makes this quite challenging [43]. Two clinical trials based in East Asia showed benefit in terms of decreased DILI by assigning isoniazid dose based on *NAT2* genotype and could be followed up in other populations [55, 56].

### 3.4 DILI Due to Other Anti‐Infective Agents: Minocycline, Nitrofurantoin, Terbinafine, Azithromycin, Amoxicillin, Trimethoprim‐Sulphamethoxazole

Genetic studies on DILI have extended beyond the common causes of this toxicity described in Sections 3.1, 3.3 to include other anti‐infectives such as minocycline, azithromycin, nitrofurantoin, trimethoprim‐sulphamethoxazole, amoxicillin and terbinafine. These drugs are each well‐established causes of DILI, but relatively few cases tend to be available for detailed genetic studies, either because the drug is not prescribed as frequently as the causative drugs discussed in Sections 3.1, 3.3 or the incidence of DILI due to the particular drug is lower. Cases of DILI due to these drugs have been included in several recent GWAS [32, 57, 58], and additional HLA genotyping by DNA sequencing has also been undertaken [59, 60, 61]. Progress has been made in detecting some specific HLA associations (see Table 2), but, in general, the findings have not been as well replicated as those described in Sections 3.1, 3.3, mainly because of limited sample availability. Associations between terbinafine DILI and *HLA‐A:33:01* [58] and minocycline DILI and HLA‐B*35:02 [63] are the most convincing of these associations, both due to the effect size (Table 2) plus the ability to detect these associations by GWAS, not simply by HLA genotyping. The terbinafine DILI association with HLA‐A*33:01 has been partly replicated by inclusion of some additional cases after the original report [58, 62]. An association reported between HLA‐DRB1*11:04 and nitrofurantoin DILI [60] did not replicate in an independent cohort of cases with a similar phenotype [64]. This likely reflects the fact that the original association relied on a few cases only with effect sizes smaller than those reported for DILI due to drugs such as AC and flucloxacillin. Amoxicillin alone sometimes causes DILI though the incidence is much lower than that seen for AC. HLA typing by imputation of genotypes in amoxicillin DILI cases found a significant association with HLA‐B*58:01 and borderline associations with HLA‐DPB1*01:01, HLA‐A*01:01 and HLA‐C*03:02 [7].

**TABLE 2:** Possible HLA associations for DILI due to anti‐infectives.

HLA allele	Drug	Allele Frequency in European DILI cases	Allele Frequency in European controls	Odds ratio (allelic) (95% confidence interval)	*p* value	Reference
A*33:01	Terbinafine	0.43	0.01	40.5 (12.5–131.4)	6.7 × 10^−10^	[58, 62]
B*14:01	Trimethoprim‐sulfamethoxazole	0.049	0.009	9.20 (3.16–22.35)	0.0003	[59]
B*35:02	Minocycline	0.08	0.003	29.6 (7.8–89.8)	2.57 × 10^−8^	[63]
B*58:01	Amoxicillin	0.07	0.01	20.7 (4.3–99.1)	0.0002	[7]
DRB1*11:04	Nitrofurantoin	0.08	0.02	4.3 (2.2–7.7)	1.2 × 10^−4^	[60]
DQA1*03:01	Azithromycin	0.29	0.11	3.4 (1.7–6.5)	0.001	[61]

*Note:* These possible associations have been reported but not yet replicated independently. Data reported are for White Europeans due to limited data available to date for other ethnicities. In the case of trimethoprim‐sulfamethoxazole‐DILI, some evidence that B*35:01 is a risk factor in African Americans has been obtained but formal statistical analysis was not reported [59].

Abbreviation: HLA, human leucocyte antigen.

In addition to the associations with specific HLA alleles, weak associations (*p* = 0.01) with *PTPN22*, a well‐replicated risk factor for AC DILI, have been detected for DILI due to sulfamethoxazole‐trimethoprim and terbinafine [32]. For amoxicillin DILI, rs1363907 in *ERAP2* increased risk of DILI in a cohort of 20 cases (*p* = 0.04); interestingly, this association originally seen for AC DILI extended to amoxicillin alone but was not seen with any other drug cause [4]. As discussed in Section 4, these additional immunogenetic risk factors seem likely to promote T‐cell responses involved in DILI, though it is also clear that they are drug‐specific and do not extend to all types of DILI showing HLA associations.

### 3.5 DILI Due to Nonsteroidal Anti‐Inflammatory Drugs (NSAIDs)

NSAIDs have been recognised as an important cause of idiosyncratic DILI for at least 40 years with early detailed surveys in relation to cases due to diclofenac, sulindac and nimesulide providing data on incidence and phenotype [65, 66, 67]. A study of DILI cases across several databases found frequent representation of NSAIDs, especially diclofenac, as causative agents with the frequency of DILI for diclofenac only slightly lower than AC and flucloxacillin [30]. Despite these valuable epidemiological surveys and the availability of sample collections more recently for genetic studies, progress on identifying genetic risk factors for NSAID DILI has been more limited than for the anti‐infective agent examples described in Sections 3.1, 3.4.

Candidate gene case–control studies on diclofenac DILI in the early 2000s suggested that a mix of pharmacogenetic and immunogenetic factors, though not HLA genotype, might be relevant to risk of DILI due to this drug but had the limitations of involving a small case‐cohort with no replication samples available and reporting relatively modest effect sizes [68, 69]. As use of GWAS approaches become more widespread, a GWAS on DILI due to the NSAID lumiracoxib, which was withdrawn during its development due to hepatotoxicity, reported a significant association with a HLA class II haplotype containing DRB1*15:01 and identical to one of the HLA risk factors for AC‐DILI [70]. This finding was unexpected due to the absence of structural homology between lumiracoxib and amoxicillin or clavulanic acid and also because the phenotype for lumiracoxib DILI was almost always hepatocellular, unlike the cholestatic or mixed phenotype typically seen for AC DILI.

The specific HLA association for lumiracoxib DILI has not been confirmed for other NSAID causes of DILI in several rounds of GWAS where diclofenac is the most common drug implicated [32, 57, 58]. Despite strong structural homology between lumiracoxib and diclofenac, these GWAS show no significant signals for DILI due to either diclofenac only or other NSAID in any gene. A study using HLA region sequencing involving 52 NSAID DILI cases from the United States (including 29 due to diclofenac) obtained a significant association with DRB1*04:03, a very rare HLA allele normally, and a parallel increase in frequency of B*35:03 [71]. Importantly, the carriage rate for each of these alleles among the DILI cases was 13% so only a small minority of cases are positive for these risk alleles. The DRB1*04:03 association could not be replicated, but the B*35:03 association was confirmed in a separate cohort of 121 European NSAID DILI cases where 56 had diclofenac DILI [71]. The frequency of B*35:03 in the diclofenac cases increased to 0.04 compared with 0.02 in controls which means that though there was MHC region significance, the contribution of this HLA allele to risk is low.

In summary, genetic risk factors for NSAID DILI are less clear than those for the anti‐infective agents discussed in Sections 3.1, 3.3 despite being common causes of DILI. There is now some evidence for a limited contribution by HLA genotype as a general NSAID DILI risk factor, but the more convincing association of DRB*15:01 with lumiracoxib DILI [70] is a special case which does not apply generally to NSAID DILI.

### 3.6 Liver Injury due to Herbal Medicine‐Induced Liver Injury (HILI)

As reviewed recently [2], liver injury due to herbal and dietary supplements (often referred to as HILI), is an important contributor to xenobiotic‐related hepatotoxicity. There are special challenges involved in studying HILI, but it is useful to consider genetic factors for HILI in parallel with those for DILI. In contrast to the situation with DILI generally where a range of HLA risk factors occur and are specific to individual drugs, HILI due to unrelated herbal medicines appears to be mainly associated with carriage of HLA‐B*35:01 (see Table 3). The clearest genetic risk factors described to date are those for HILI due to green tea and *Polygonum multiflorum* where studies in the United States and China both reporting an association with HLA‐B*35:01 when HLA genotype was investigated directly as a risk factor [72, 74]. A recent case report describes monozygotic twins positive for B*35:01 who developed liver injury at different times following consumption of a nutritional supplement known as Hydroxycut which contains green tea extract provides additional evidence of the HLA‐HILI association [73] A small study on HILI due to turmeric, based in the United States, reported a similar association with HLA‐B*35:01 [75]; there is also a possible B*35:01 association with HILI induced by *Garcinia cambogia*, an agent used to aid weight loss [76]. Finally, HLA‐B*35:01 was more common in cases of HILI due to Japanese Kampo medicines, whose composition is different to the green tea and polygonum preparations [77]. In view of the complexity of herbal products in terms of content, the finding of a similar risk factor for five different supplements is a striking finding, especially as the reports indicate that 90% of well‐adjudicated HILI cases due to green tea and 45% due to polygonum carry this allele. HLA‐B*35:01 has been shown to be a strong risk factor for autoimmune hepatitis in Han Chinese, though not Europeans, which may reflect previous exposure to viral peptides in the Han Chinese [78]. However, it appears that the B*35:01 association with HILI extends to Europeans. The causes of HILI seem diverse chemically, but the case phenotype seen in the B*35:01‐HILI association is almost entirely hepatocellular. Interestingly, there is a suggestion that B*35:01 is also a risk factor for DILI due to sulfamethoxazole‐trimethoprim in African Americans but not European Americans (see Table 2). This needs confirmation in a larger cohort.

**TABLE 3:** Herbal medicines causing hepatotoxicity for which an association with HLA‐B*35:01 has been reported.

Preparation	Study location	Reference
Green tea	USA, Iceland	[72, 73]
*Polygonum multiflorum*	China	[74]
Turmeric	USA	[75]
*Garcinia camborgisa*	USA	[76]
Kampo medicines	Japan	[77]

Abbreviation: HLA, human leucocyte antigen.

### 3.7 Immune‐Mediated DILI due to Indirect Hepatotoxicity

As drug treatments evolve towards increased use of biologics and personalised approaches, a new form of hepatotoxicity which is distinct from idiosyncratic DILI has been proposed [1, 79]. This involves indirect toxicity where the DILI results from drug action, not inherent hepatotoxicity. This evolving area is important, especially because the injury occurs more frequently than idiosyncratic DILI. Typically, indirect hepatotoxicity involves a reaction to a medication class rather than a more specific individual drug and may occur months after first exposure to the agent. Drugs causing this type of toxicity include interferon, tyrosine kinase inhibitors and monoclonal antibodies to proteins such as tumour necrosis factor‐alpha or checkpoint proteins. The disease typically shows autoimmune features.

Data on genetic factors affecting susceptibility to indirect hepatotoxity are limited, but there are a few reports relating to interferon beta [80], infliximab [81] and checkpoint inhibitors [82] that have reported significant findings. The study on interferon beta involved GWAS on 56 cases and found a genome‐wide significant signal adjacent to the interferon regulatory factor 6 (IRF6) gene but no HLA signal [80]. On the contrary, a study on 16 cases of DILI due to infliximab found a significant association with the rare HLA allele B*39:01 [81]. This allele was carried by four cases only, and no replication was possible. For the checkpoint inhibitors, 57 cases with toxicity due to several different drugs were analysed by sequencing‐based HLA genotyping and candidate gene studies on selected candidates relevant to these drugs [82]. There is considerable heterogeneity with respect to tumour type and drug treatment in this cohort, but no HLA alleles, including those associated with autoimmune hepatitis, showed an association with DILI. For the selected candidate genes, certain polymorphisms in five different genes relevant to either host immune response or other functions showed an increased risk for DILI. None of these variants had been associated with DILI due to any drug previously.

As reviewed recently, there are a relatively large number of reports describing liver injury after vaccination with COVID‐19 mRNA vaccines [1]. This type of reaction to mRNA vaccination is very rare and was suggested initially to be a form of autoimmune hepatitis. However, RNA sequencing was performed on liver biopsies to compare gene expression in the vaccine‐induced liver injury with autoimmune hepatitis [83]. There were similarities in the expression of some genes between both sets of biopsies, but samples from vaccine‐induced cases showed differential expression of genes relevant to mitochondrial metabolism and oxidative stress responses not seen in the autoimmune hepatitis cases. This vaccine‐induced injury was therefore considered to be more closely related to drug‐induced autoimmune‐like hepatitis or possibly the ‘indirect hepatotoxicity’ defined recently. In a limited genetic study on 16 well‐adjudicated vaccine‐related DILI cases, no evidence for altered frequency of HLA alleles associated with autoimmune hepatitis was obtained but the polymorphism in *ERAP2* previously reported as a risk factor for AC‐DILI (Section 3.1) and a linked haplotype involving the adjacent *ERAP1* gene occurred at a significantly increased frequency in these cases [84]. These data are of a preliminary nature, but the *ERAP* association is consistent with an altered host immune response in this rare event.

### 3.8 General Non‐drug‐Specific Factors

It was originally assumed that by performing genetic studies, common risk factors for idiosyncratic DILI generally could be identified, though there was also general acceptance that some factors such as those relevant to disposition of drugs would be drug‐specific. As described in Sections 3.1, 3.7, success in identifying risk factors common to more than one form of DILI has been limited. HLA has emerged as playing a more important role than was expected originally, but different HLA genes and alleles contribute to the different forms. A small number of common risk factors has emerged including: (i) HLA‐DRB1*15:01 as a risk factor for DILI due to both AC and lumiracoxib, (ii) HLA‐B*35:01 as a risk factor for HILI caused by several different herbal medicines, (iii) rs2476601 in *PTPN22* as a strong risk factor for AC DILI but also relevant to risk of sulfamethoxazole‐trimethoprim and terbinafine DILI, (iv) *ERAP2* rs1363907 appears relevant to risk of AC DILI, amoxicillin DILI and vaccine‐related DILI.

As an alternative to a single risk factor for DILI, can a PRS be developed as has already been achieved for a number of complex diseases? [85] Section 3.1 describes the development of a PRS for AC DILI [4], but this PRS is specific for this form of DILI and cannot be usefully applied to DILI caused by other drugs. A different approach using more global GWAS data has led to the development of three very complex but potentially valuable PRSs for DILI [86]. These scores were developed originally because of a problem with DILI during a phase 3 clinical trial of the drug fasiglifam, which led to a plan to use existing GWAS data from DILI cases due to a range of different drugs to help identify the genetic risk factors responsible in a group of 43 fasiglifam DILI cases. Published GWAS data for approximately 4 million single‐nucleotide polymorphisms (SNPs) [58] was used to develop scores with approximately 28 000 SNPs that showed *p* values < 0.5 for DILI and were situated in the approximately 15 000 genes expressed in human hepatocytes. The 28 000 SNPs were selected after ‘clumping’ of SNPs that were in linkage disequilibrium. The GWAS had considered hepatocellular DILI and cholestatic/mixed DILI separately, and this allowed the development of three separate PRS‐one for all DILI, one for cholestatic/mixed and one for hepatocellular. All three scores were then applied to the fasiglifam DILI cases and controls. The PRS for cholestatic/mixed DILI was significantly higher for the fasiglifam cases compared with controls. The PRS were also applied to a separate cohort of AC and flucloxacillin DILI cases that had not been included in the original study. The cholestatic/mixed PRS also showed significant differences in scores between cases and controls for both drugs separately or combined. Pathways represented by the PRS were also investigated, and for the cholestatic/mixed PRS, oxidant‐induced survival, unfolded protein response and mitochondrial activity pathways were significantly enriched. Though the significant signals detected for DILI by GWAS to date do not feature in these pathways, there is some limited evidence of the relevance of the pathways from both candidate gene studies and cell‐based analyses. The complexity of these PRS means routine clinical use is not feasible, but they may be of value during drug development, especially if evidence of a potential DILI risk needs further investigation.

## 4 Underlying Mechanisms for DILI: Increased Understanding From Genetic Studies

Underlying mechanisms for development of idiosyncratic DILI remain poorly understood though progress on developing cell‐based models has been made, with some approaches incorporating genomic data. For certain other types of non‐DILI adverse drug reactions where HLA associations have been described, studies involving either isolated T cells or peripheral blood mononuclear cells cultured with autologous antigen‐presenting cells and the drug causing the adverse reaction have been used to confirm the importance of T‐cell responses in the toxicity. Such studies were performed originally to determine the mechanism underlying the abacavir hypersensitivity association with HLA‐B*57:01 and were subsequently used to study other associations such as that between carbamazepine‐induced skin rash and HLA‐B*15:02 [87]. As positive end‐points, cell proliferation specific to cells from donors with the at‐risk HLA genotype and production of inflammatory cytokines have been used. In relation to DILI, this approach was first used to show proliferation in T cells from donors positive for HLA‐B*57:01 cultured with flucloxacillin [8]. Broadly similar cell‐based studies have shown HLA‐specific T‐cell responses to both amoxicillin and clavulanic acid for the genotypes associated with AC DILI [3]. For the HLA‐B*35:01 risk factor for HILI (Section 3.6), T‐cell proliferation in individual blood donors with the B*35:01 genotype but naive to herbal medicines has been demonstrated by culturing white cell extracts with a component of green tea extract [88]. These studies all confirm a role for the particular HLA protein encoded by the DILI‐associated HLA allele in presenting peptides inappropriately to T cells in the presence of drug and promoting T‐cell proliferation. It is assumed but has not been shown directly that T cells within the liver proliferate and cause liver injury during DILI.

Though animal studies are generally of limited value as models for idiosyncratic DILI, a recent study involving HLA‐B*57:01 transgenic mice has shown a HLA‐B*57:01–dependent T‐cell reaction to flucloxacillin via CD8‐positive T cells is limited by the presence of CD4^+^ cells and PD‐1 expression. Blocking the CD4‐positive cells and knocking out PD‐1 led to hepatic changes but not actual DILI in the drug‐exposed animals [89]. This is not dissimilar to the response to flucloxacillin in humans where only 0.1%–0.2% of those carrying HLA‐B*57:01 and exposed to flucloxacillin develop DILI with clearly other factors being required to induce disease. As described in Section 3.2, there is some evidence that increased age and high drug levels for longer periods are a risk factor in flucloxacillin DILI [39] but additional genetic risk factors are still understood poorly.

For both flucloxacillin and AC DILI, data describing direct toxic effects in cultured human hepatocytes involving oxidative and endoplasmic reticulum stress together with effects on other cellular processes such as alterations in bile acid homeostasis with flucloxacillin, amoxicillin and clavulanic acid when very high concentrations of the drugs are added provide some additional insights into likely additional cellular risk factors [90, 91, 92]. These findings are consistent with overall susceptibility involving multiple signals which would agree with the findings for the pathways underlying the complex PRSs discussed in Section 3.8.

In parallel with these cell‐based approaches, molecular modelling studies involving either the DILI‐causing agent or a metabolite and the particular HLA risk factor protein have indicated a direct interaction between drug or metabolite and the HLA molecule similar to that already established to occur for B*57:01 and abacavir [87]. These studies suggest an ionic interaction between HLA protein and drug may occur for minocycline and trimethoprim‐sulfamethoxazole [59, 63]. Earlier studies suggest that flucloxacillin does not bind directly to HLA‐B*57:01 and is not similar to abacavir in that respect [93]. There is now considerable evidence that flucloxacillin binds strongly to cellular proteins in vitro and in vivo, and it appears more likely that flucloxacillin‐modified peptides are being presented inappropriately to T cells [94, 95].

For DILI causes such as isoniazid, where a role for HLA genotype in susceptibility has not been established (Section 3.4), the underlying mechanism for DILI is less clear than the examples discussed above. However, as discussed in detail recently [96], failure to detect an HLA association in DILI does not mean that the mechanism is not immune‐related and that it purely relates to metabolic idiosyncrasy. Isoniazid, when oxidised, binds covalently to a large number of cellular proteins [97], and this may also be the case for its major metabolites acetylhydrazine and hydrazine [96]. This widespread covalent protein binding may result in many drug‐modified peptides which could cause an extensive immune response involving multiple HLA proteins [96]. ‘Ultraslow acetylators’ who appear to be at increased risk of isoniazid DILI (Section 3.4) could show an altered pattern of protein modification due to either a higher cellular level of isoniazid or accumulation of hydrazine or possibly both. This may provoke a stronger immune response resulting in cellular damage. Although mitochondrial toxicity has been proposed as an alternative mechanism for isoniazid DILI [98], some recent reports suggest that this is unlikely [96, 99].

## 5 Conclusions

Idiosyncratic DILI, together with the closely related indirect DILI, continues to be an important aspect of hepatology, especially as new drug causes of this toxicity are still emerging. The considerable developments in genomics in the last 20 years have enabled some genomic risk factors for DILI to be identified, though what has been discovered to date is limited and unlikely to be a means of eliminating idiosyncratic DILI generally. Most of the genomic risk factors identified to date are drug‐specific which means genotyping for these factors should be a useful means of diagnosing DILI and preventing dangerous re‐exposure of the patient to the causative agent. However, genotyping prior to drug prescription is not currently realistic as the proportion of patients carrying the at‐risk genotypes is low. It also remains possible that rare genetic variants that are only detectable by genome sequencing contribute to DILI susceptibility, but, to date, this aspect remains unexplored.

As patterns of drug prescription change, causes of DILI, especially in a hospital setting, have also changed with a recent report showing anticancer drugs to be the most common cause of DILI in a tertiary referral centre [100]. These newer causes of DILI are likely to have a variety of different genetic risk factors which are currently not well understood, as discussed in Section 3.7. The more traditionally seen types of idiosyncratic DILI, especially those due to anti‐infective agents, are likely to continue to occur as the only means of preventing these reactions currently would be removal of the causative drugs from the market which would be problematic for both patients and prescribers. There is now better awareness of the risks of DILI among drug developers and regulators worldwide, and this makes it less likely that new ‘small molecule’ drugs that cause idiosyncratic DILI will be licensed as current knowledge on DILI, including genetic risk factors, is used to inform drug development. Biologic agents and vaccines seem likely to be important causes of DILI in the future, and further genomic studies on this aspect are needed.

## Conflicts of Interest

The author declares no conflicts of interest.

## Data Availability

The author has nothing to report.
